# Reinforced Spun Concrete Poles—Case Study of Using Chemical Admixtures

**DOI:** 10.3390/ma13020302

**Published:** 2020-01-09

**Authors:** Romualdas Kliukas, Arūnas Jaras, Ona Lukoševičienė

**Affiliations:** Department of Applied Mechanics, Faculty of Civil Engineering, Vilnius Gediminas Technical University, Saulėtekio al. 11, LT-10223 Vilnius, Lithuania; romualdas.kliukas@vgtu.lt (R.K.); arunas.jaras@vgtu.lt (A.J.)

**Keywords:** spun reinforced concrete, chemical admixtures, production technology, spinning equipment, poles, overhead electric power lines, mechanical properties

## Abstract

The current research paper is focused on the experimental investigation of features of chemical admixtures (superplasticizers C-3, ‘Dofen’ and formaldehyde resin ACF-3M) utilizing in reinforced spun concrete structures. For the sake of comparison, the results of studying the effects of chemical admixtures on physical and mechanical properties on vibrated and spun concrete are provided. As a separate part of spun concrete products, the supporting poles of overhead power lines are introduced. The results obtained indicate, that the positive effect of chemical admixtures for spun and vibrated concrete is most pronounced at an early age of concrete. The effective amount of chemical admixtures for spun concrete is 0.15% of cement mass when formaldehyde resin ACF-3M and 1% of cement mass when superplasticizers C-3 and ‘Dofen’ are used. Moreover, the brief review about the reinforced spun concrete members is provided.

## 1. Introduction

**Figure 1:**
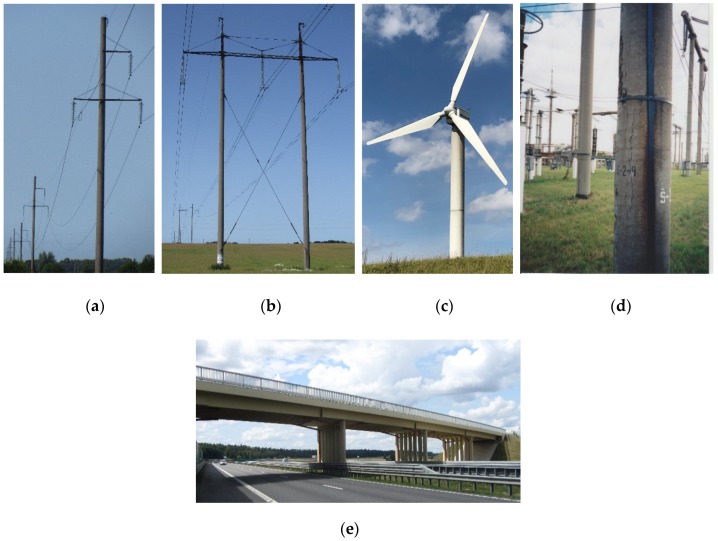
Using of the reinforced spun concrete members: (**a**) monopolar power transmission lines; (**b**) bipolar power transmission lines; (**c**) wind turbine towers; (**d**) electric power distribution substations and (**e**) poles of overbridges.

In transport, power engineering, industrial and other constructions, prefabricated reinforced concrete structures are often used. These structures include spun reinforced concrete members. These members are the foundation poles, lampposts, the supporting poles of overhead power lines, power distribution structures, the supporting structures of railway power lines, the structural elements of bridges, viaducts and seaports, as well as low power wind turbine towers and other supporting structures for general use, etc. Some typical cases of using reinforced spun concrete members in engineering practice are given in Figure 1.

Due to their construction peculiarities, most of spun concrete members are of an annular cross section. The structures with this cross section are lightweight, while their strength and stiffness are the same in all cross section directions, their bending strength is very high and they can withstand higher loads than reinforced members made by using vibration.

In 1907, in Europe (Germany), the first centrifuges for making hollow spun concrete elements were made. This modern technology of spun concrete production was first used for making the supports for overhead power lines [1]. Later, this advanced technology was used in other countries (e.g., France and Belgium) for making poles, pressure pipes, columns, beams and other members and structures.

In the former Soviet Union, after World War II, the production of spun reinforced concrete supporting poles for power lines of 110, 220, 300 and 500 kV grew tenfold [2] when the electrification of the country began.

In the USA, the use of spun reinforced concrete members was also closely associated with the electric power companies, but began sometime later, in about 1960. The production and use of these structures in power transmission systems had increased considerably in this country since the mid-1970s. The “state-of-art” of the technology of spun concrete poles for applying the electrical transmission structures in USA was described by W. Oliphant and C. J. Wong [1].

At that time, rapid industrial development increased the need for electric power and, consequently, intensified the construction of overhead power lines. Due to their great strength and small weight, as well as durability, spun reinforced concrete members were widely used for electric power transmission purposes. As a result, most of the European countries and the USA unified the supporting poles used in electric power transmission, while their technology was greatly improved by using the innovative and most effective equipment [3,4,5].

Further advance of spun reinforced concrete members’ production and use, which was closely associated with the improvement of their production technology and widening the area of their application, took part in the last decade of the 20th century. At that time, the plants producing reinforced concrete members were reconstructed by introducing more advanced and faster production lines, though the production process based on centrifugation remained the same. The survey of the development of spun prestressed concrete poles (past, present and future) is presented by F. Fouad, D. Sherman and R. Werner [6].

Now, large numbers of spun reinforced concrete structures, particularly those used for making the supporting poles of overhead power lines, are produced in Canada, USA, France, Germany and in some republics of former Soviet Union [7,8,9].

In Lithuania, the studies of physical and mechanical properties on reinforced spun concrete have been performed since 1959 at the base of the former Vilnius Civil Engineering Institute (now, Vilnius Gediminas Technical University). These studies mostly were focused on experimental and theoretical research on the supporting poles of overhead power lines and communication systems [10,11,12,13,14].

Recently new materials for reinforced concrete structures’ production, such as fine aggregates and various chemical admixtures, which improve the properties of concrete (increase the plasticity of concrete mixes, accelerate or decelerate concrete hardening, decrease its porosity, etc.) are used. Moreover, stronger steel reinforcement combined with composite non-metallic reinforcement (e.g., carbon and glass fiber), was also used for making hybrid (tubular steel and spun concrete) members (poles), etc. [15,16,17,18].

Current investigation is focused on the features of chemical admixtures (superplasticizers C-3, ‘Dofen’ and formaldehyde resin ACF-3M) utilizing in reinforced spun concrete structures. The results of experimental study about the effects of plasticizing chemical admixtures on physical and mechanical properties on vibrated and spun concrete members are provided. Moreover, the brief review about the reinforced spun concrete members is provided.

## 2. The Reinforced Spun Concrete Members: Manufacturing and Features

Spun reinforced concrete structures are made by using centrifugation equipment based on the principle of spinning the product being formed. In the process of production, concrete mixture and reinforcement placed into steel molds are spun in them, following a particular operation mode.

**Figure 2:**
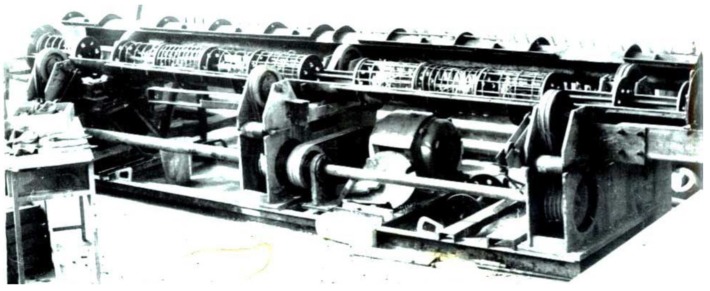
The integrated roller and belt-driven type centrifugal machine and an open semi-mold with the reinforced carcass.

Roller-type and belt-drive type, as well as axial centrifuges are used in technological lines. As an intermediate option, the integrated roller belt-drive type centrifuges can be used (Figure 2), though roller-type centrifugation equipment is most commonly used in engineering practice.

Due to the production peculiarities in spinning, the members of annular cross section are mostly used. However, they can also be of other forms, e.g., rectangular, square, hexagonal and octagonal forms, and usually have a cylindrical opening in the center.

The centrifuging process is performed in stages by gradually varying (controlling) the spinning rate of a steel mold filled with a concrete mix as follows:

1. Speed 1. A tubular element is formed at the specified spinning rate of the steel mold, not allowing the concrete mix to run out. The spinning time is *t* = 3–4 min.
2. Speed 2 and 3. Concrete compacting and exceed water removing are begun. The spinning rate of the mold is increased until the design rate. The spinning time is *t* = 1–2 min.
3. Speed 4. The concrete mix is compacted at the specified design spinning rate. The spinning time is *t* = 10–15 min.

Each stage is characterized by the particular spinning speed of the product, which depends on the product’s dimensions and the time required for maintaining the selected speed. While the spinning time of a reinforced concrete member in the steel mold depends on the initial water–cement ratio ${\left(W/C\right)}_{in}$ and the pressing pressure, caused by the centrifugal forces, depending on the spinning rate of the centrifuge and the product’s dimensions (diameters).

Generally the production of spun reinforced concrete members depends on a number of technological factors as follows:

1. Mode of centrifuging (e.g., the moment of achieving the highest rate of the mold’s spinning rate and its duration);
2. The maximum pressing pressure in the mold, depending on the spinning rate of the mold and the product’s dimensions;
3. The amount of water, remaining in the unhardened concrete after centrifugation;
4. The size and the form of the elements (e.g., a cylindrical or conical form);
5. The number of the placing operations of the concrete mix in the mold.

The quality of the spun concrete largely depends on the time when the maximum spinning rate of the steel mold could be achieved and the time of this mode of operation. When the spinning rate of the mold is further increased, the concrete, acted upon by the centrifugal forces, is closely retained against the mold’s walls. As a result, heavier concrete mix components move closer to the mold’s walls, while light components, such as water and contaminants are pressed out to the inner surface of the product, i.e., towards the rotation axis. The inner part of the product becomes smooth and the water found on it flows out through the holes at the ends of the spun mold. If the maximum rate of the mold is reached too quickly due to rapid pressing out of the water from the concrete mix, the grout can be washed out. Then, the inner surface of concrete can become too coarse or ‘a heavy’ aggregate fraction (crushed stone) can be observed on the surface of the exterior layer.

**Figure 3:**
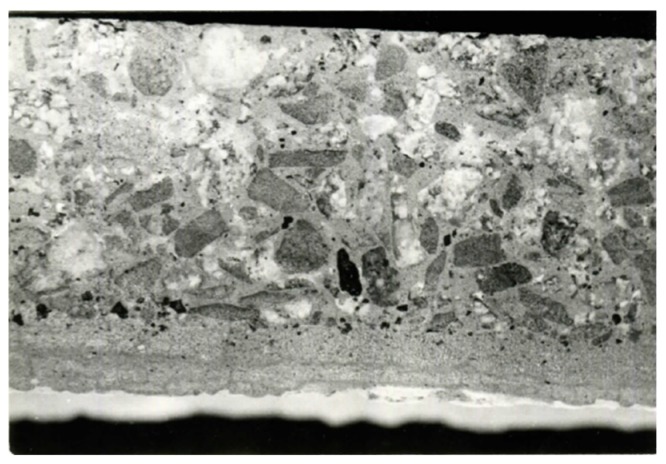
The structure and texture of an annular spun concrete member (the view of a longitudinal section).

Compared to the similar cases of vibrated concrete, spun concrete has its specific structure and texture. It has non-uniform structure and texture per wall thickness of the element [19,20,21]. After centrifugation, the outer surface of the element made according to the specified technology, which was closely retained against the mold’s walls, is dense and covered with a thin cement layer. The concrete near the inner surface of the product has more grout than in other areas of the cross section because the grout was pressed out from the product together with water. Though crushed stone distributed almost evenly through the wall, however, in centrifuging, larger parts of this material were concentrated on the outer part of the product, decreasing its deformability and increasing its resistance to atmospheric effects and impacts, as well as strengthening and compacting the area where the reinforcement was found. Therefore, concrete is not uniform strength through the wall thickness [19,21]. The detailed view of the described variation of spun element’s structure in the radial direction of its cross section is given in Figure 3.

The spun concrete members are acted upon by the centrifugal force, which is strongly pressing the concrete mix near the mold’s wall. The pressure compacting the mix, which is caused by the action of the centrifugal force, is not distributed uniformly. This results in a larger water–cement ratio (W/C), and the porosity can be observed in the layers found closer to the rotation axis of the mold, i.e., closer to the inner surface of the product (Figure 3). However, the largest amount of water is flowed out from the outer layers of the product, which are acted upon by the highest pressing force. In fact, around the inner product’s surface, where the pressing force is about zero, almost no water is removed from the concrete mix.

In the process of centrifuging a concrete mix, water filtration from one layer to another causes the formation of microcapillaries in the concrete, which are directed in the radial direction from the outer layer of the mix to its inner layer. These capillaries are combined, while the element’s interior walls have the capillaries decreasing the strength of the concrete mix. Their number depends on the initial water–cement ratio ${\left(W/C\right)}_{in}$. The smaller this ratio, the denser and more uniform the concrete structure is. It should be noted that chemical admixtures could decrease the initial water–cement ratio, not decreasing such concrete properties as workability and slump.

The question arises, what spinning rate and pressing pressure associated is optimal. The tests made by [22] allowed the author to state that the increase of the pressure to 0.1 MPa greatly changes the residual water–cement ratio. However, when the pressure is increased from 0.1 to 0.15 MPa, the residual water–cement ratio actually remains unchanged. Therefore, it was assumed, that the optimal pressure is 0.1–0.12 MPa. Under such pressure and with the mold’s spinning time of about 10 min, the residual water–cement ratio ${\left(W/C\right)}_{resid}$ remains optimal, thereby ensuring normal concrete hardening.

The pressing pressure of a concrete mix can be expressed by the Equation (1) [10]:

(1) $$p=\frac{\gamma \;\omega^{2}}{2g}\left(r_{2}^{2}-r_{1}^{2}\right),$$

where: γ—force of gravity of the concrete mix ($\mathrm{kN}/m^{3}$); ω—angular velocity of mold (rev/min); g—gravity acceleration (m/s^2^) and $r_{1}-r_{2}$—internal and external radii of annular cross section, respectively (m).

According to I. Achverdov [23], under the optimal pressure ($p_{opt}$) the amount of the flowed out water should be such that the average residual water–cement ratio ${\left(W/C\right)}_{resid}$ of the grout should satisfy its normal density coefficient value. The studies of I. Achverdov have shown that the pressure $p_{opt}=0.065\;\mathrm{MPa}$ is optimal for heavy concrete. However, it should be noted that in the production of small cross section elements (diameter up to 300 mm), it is often difficult to achieve the optimal pressure ($p_{opt}$) because the required spinning rate of this steel mold is difficult to achieve in the process of centrifuging the concrete element. The practice of experimenting has shown that under long-term pressing pressure p≥0.02 MPa, the concrete is well compacted.

According to M. A. Reut [2], the strength of the concrete mix made of cement, sand and broken granite can be defined approximately, depending on its water–cement ratio as in Equation (2):

(2) $$f_{c}=\frac{A_{c}\;\left(C-0.5\;W\right)}{W\cdot K},$$

where: $f_{c}$—concrete compressive strength after 28 days of hardening (MPa); $A_{c}$—cement activity (MPa); C—the amount of cement for 1 m^3^ of concrete (kg); W—the amount of water for 1 m^3^ of concrete (kg) and K—the coefficient, depending on the kind of aggregate (crushed stone, K=2).

The verification study, conducted by the author of this article (R. Kliukas) confirmed the appropriateness of the above formula. The experiments were performed under factory conditions, to compare the strength of the vibrated and spun concrete. For this purpose, the concrete mix, consisting of $1080\;\mathrm{kg}/m^{3}$ broken granite (5–20 mm-large fraction), $680\;\mathrm{kg}/m^{3}$ of sand (the gradation factor of 3.06), $450\;\mathrm{kg}/m^{3}$ of cement (cement activity $A_{c}=51.6\;\mathrm{MPa}$) and $198\;\mathrm{kg}/m^{3}$ of water was used. The initial water–cement ratio ${\left(W/C\right)}_{in}=0.44$ and the residual ${\left(W/C\right)}_{resid}=0.32$. The spinning rate ω=115−390 rev/min, pressing pressure p=0.1−0.2 MPa and the total spinning time t=17 min.

Using a special prism shaped forms (in addition to the main mold of the product), seven vibrated concrete prisms and 12 spun concrete prisms, as well as a 22 m-long cylindrical pole with 560 mm outer diameter, were made of this mix. All the products were hardened by heating them in the induction furnace.

The prism specimens were tested by loading them with a short-term axial compressive load. The experiment has shown that after 28 days of hardening the mean strength of the vibrated prisms’ concrete was $f_{c,\;v}=42.9\;\mathrm{MPa}$, while that of the spun prisms’ concrete was $f_{c,\;c}=69.1\;\mathrm{MPa}$.

Calculating by the above mentioned Formula (2), the strength of the vibrated and spun concrete prisms was obtained $f_{c,\;v}=45.7\;\mathrm{MPa}$ and $f_{c,\;c}=67.7\;\mathrm{MPa}$ respectively.

Thus, the obtained strength of the spun concrete prisms is by 1.61 times (61%) higher than the strength of the vibrated concrete prisms, while the results obtained allow the researchers to confirm the suitability of Formula (2) for determining the relative prism strength of vibrated and spun concrete.

The structural members of reinforced spun concrete have multiple advantages. As mentioned above, due to the specific method of production (centrifugation) the concrete in the rotated mold is acted upon by centrifugal forces and pressed against the mold walls. Thus, the concrete is compacted greatly and is much (1.3–1.8 times) stronger than the concrete of the same composition, compacted using other methods [13,19,20,21].

The spun concrete structures of annular cross section ‘work’ well under torque as opposed to the elements of rectangular or double tee cross sections [24]. Furthermore, reinforced spun concrete elements are massive, which makes them more resistant to dynamic loads (e.g., those occurring during car crashes) [25] and to repeated loads (e.g., wind gusts) [26]. The vibration analysis of transmission poles made of spun concrete was performed by K. Dai and S. Chen [27].

Since the reinforced spun concrete members are usually of annular cross section, the moment of inertia of their cross section is 3–6 times larger than that of solid elements of circular cross section of the same area. Therefore, structural members made of reinforced spun concrete have much higher strength and stiffness under bending and twisting loads, while their strength under compressive loads is also significantly better [24,28,29,30]. This allows for producing lighter and cheaper structures (with a smaller cross section area). The research shows that the elements of reinforced spun concrete are 30–40% cheaper than the same elements made of vibrated concrete because the amount of reinforcing steel and energy used are reduced by 25% and 15%, respectively, while labor costs are decreased by 56% [10]. The special study for improving durability and performance of spun concrete members is presented by W. Digler, A. Ghali and M. Rao [31].

Reinforced spun concrete members of annular cross section also have other benefits related to their structural use. For example, they are ideally suited for supports of overhead power lines (Figure 1a,b) since they are resistant to changing atmospheric conditions and are durable, while their resistance to mechanical actions is the same in any direction of the cross section. Spun concrete has minimal water permeability, which means that long-term use of its products outdoors (e.g., the supports of overhead power lines) has a negligible effect on its strength. The results obtained in testing the state of overhead power line supports, which are more than 30 years old, in Lithuania, demonstrated that spun concrete members are significantly more durable and reliable when compared to the members of vibrated concrete [14]. The benefits of spun prestressed concrete poles of overhead electric power lines in respect of wooden and steel poles are presented in [32].

## 3. The Experimental Study of the Effectiveness of Chemical Admixtures in Concrete Mixes

Various admixtures are added to concrete mixes to improve the properties of concrete products. They are divided into mineral and chemical admixtures. Different countries (EU member states, USA and Russia) have their own standards and regulations, as well as their classifications and use [33,34,35].

Generally, chemical admixtures can controlling physical and mechanical properties of concrete mixtures (plasticizes, reducing water and cement requirement, etc.), controlling the kinetics of hardening of concrete mixtures (accelerators, retarders, etc.) or controlling physical and mechanical properties of the concrete stone (increasing its strength, corrosion resistance, water tightness, porosity, etc.).

It should be noted that there are a considerable amount of papers on the effect of various chemical admixtures on physical and mechanical properties of vibrated concrete [12,36,37,38]. However, the research on the properties of reinforced spun concrete made of cement mixes with chemical admixtures is significantly less.

This paper analyzed the effect of plasticizing chemical admixtures on physical and mechanical properties of the members of reinforced spun concrete. The tests under laboratory and factory conditions, for studying the effect of chemical admixtures on the physical and mechanical properties of vibrated and spun concrete, were performed. Superplasticizers, C-3 and ‘Dofen’, as well as the formaldehyde resin ACF-3M, were used in testing. The selected chemical admixtures are perfect for cold climate countries and are widely used in concrete factories.

The superplasticizer C-3 is a synthetic product based on the sulphonated naphthalene formaldehyde resin. The superplasticizer ‘Dofen’ is an oligomeric compound based on a sodium salt and naphthalene sulfonic acid. The acetone–formaldehyde resin ACF-3M is a condensation product of acetone and formaldehyde.

Research on spun polymer cement concrete in Lithuania was initiated by R. Garalevičius [11]. He demonstrated that the amount of chemical admixtures (water-soluble resins), which give concrete the maximum strength under tensile and compressive loads makes only 1.5–2% of cement mass. This allows us to achieve the same concrete strength as in the case of regular compaction and also use less cement. Polymeric admixtures improved concrete slump and workability and in turn allowed to reduce water requirement for the concrete mix.

Researchers A. Kudzys, R. Vadlūga, V. Drobelis and R. Kliukas carried out the study on vibrated concrete mixes, which included chemical admixtures C-3, ‘Dofen’ and ACF-3M [12]. Here 24 vibrated prism specimens including admixtures and eight control specimens, made under laboratory conditions, were subjected to the action of the short-term axial compressive load. The statistical analysis of experimental data, evaluated by average, standard deviation (SD) and coefficient of variation (CV) is presented in Table 1.

The tests have shown that the above-mentioned chemical admixtures allow for reducing the water–cement ratio (W/C) in concrete mixes. This leads to the increase in the density of the hardened concrete stone, as well as its water tightness, resistance to aggressive environments and strength.

The effect of chemical admixtures on the physical and mechanical properties of concrete was evaluated using the dimensionless coefficient α, presented the ratio as following Equation (3):

(3) $$\alpha =s_{c,i}/s_{c,o},$$

where $s_{c,i}$ and $s_{c,o}$ are the criteria for the physical and mechanical properties of the concrete with and without admixtures, respectively.

**Figure 4:**
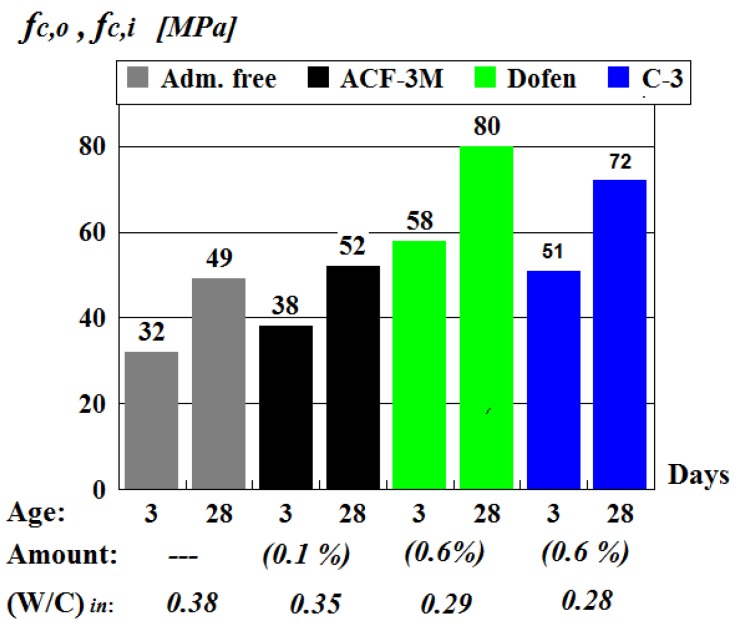
The effect of chemical admixtures on the prism strength of vibrated concrete.

**Figure 5:**
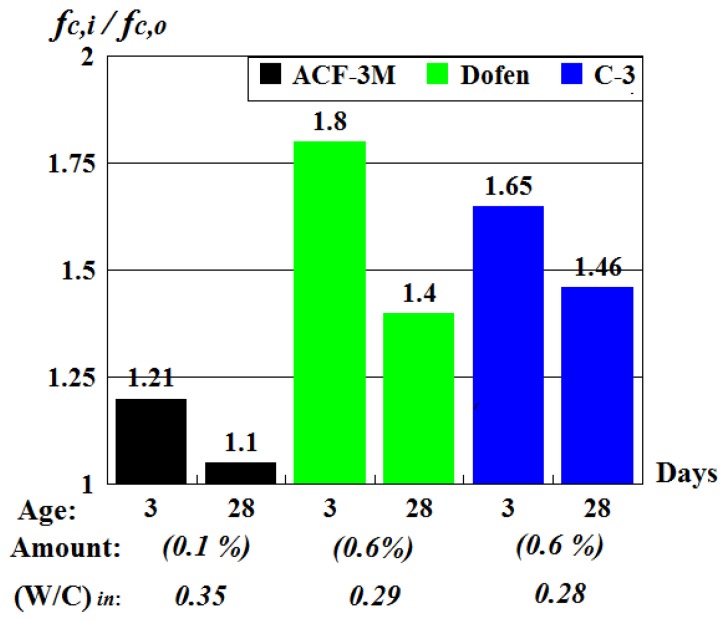
The ratios of prism strengths of vibrated concrete without ($f_{c,o}$) and with ($f_{c,i}$) admixtures.

The results obtained for vibrated concrete by testing the vibrated control cubes under laboratory conditions are given in Figure 4 as the absolute strength values of concrete without ($f_{c,o}$) and with ($f_{c,i}$) chemical admixtures, while in Figure 5 they are presented as dimensionless coefficients α, describing the admixture’s effectiveness.

The data presented in the Figure 4 confirmed that using chemical admixtures, such as superplasticizers C-3 and ‘Dofen’, as well as formaldehyde resin ACF-3M, was an effective way of improving the mechanical properties of vibrated concrete. However, it should be noted that superplasticizers C-3 and ‘Dofen’ were more effective than formaldehyde resin ACF-3M. For example, the prism strength of concrete made of the mixtures including ‘Dofen’ exceeded the strength $f_{c,o}$ of regular vibrated concrete by 40–80%, while the prism strength of concrete made of a mixture including the resin ACF-3M exceeded the value of $f_{c,o}$ only by 5–20%.

It can be seen from Figure 5 that the effectiveness of admixtures was the highest when the concrete was in its early age. This is explained by the fact that admixtures have a twofold effect on physical and mechanical properties of vibrated concrete:

1. Their use allows for decreasing the water requirement of a concrete mix without negatively affecting its workability. As a result, concrete with higher density could be obtained.
2. They facilitate more rapid formation of the concrete structure during its curing, thereby improving physical and mechanical properties of concrete at the stage of its production.

During the curing of vibrated concrete structural formation of the cement stone was slower. Due to this, it was more pronounced at the subsequent stages of concrete ageing. Therefore, the use of chemical admixtures in the production of vibrated concrete products allowed for not only significant saving of cement, but also the reduction of the curing time of concrete. This, in turn, resulted in energy savings, accelerated the turnover of steel molds and reduced the labor costs at concrete plants.

It was determined that the most effective and optimal admixture quantity (in percent of cement mass) for a vibrated concrete mix used in concrete production was 0.1% for formaldehyde resin ACF-3M and 0.6% for superplasticizers C-3 and ‘Dofen’.

The results of researches [11,12], inspired the authors to use these admixtures for the production of spun concrete. Eighty four concrete specimens of annular cross section with the diameter of 560 mm, the height of 550 mm and the wall thickness of 50–95 mm were produced to study the effectiveness of chemical admixtures in spun concrete. For concrete specimens, admixtures were added into concrete mixes in the following quantities: formaldehyde resin ACF-3M—0.15% and superplasticizers C-3 and ‘Dofen’—1.5% of cement mass because, as mentioned above, 25% of the admixtures were removed together with slurry. The specimens were produced by single-layer centrifugation in a roller-type centrifuge under factory conditions.

The centrifugation process began with distributing the concrete mix over an annular cross section. Therefore, the initial spinning rate of the centrifuge was about 100 rev/min. It should be noted that at the lower rate of spinning, the centrifugal force can be smaller than the force of gravity, while the local slides of the concrete mix are possible. Then, the spinning rate was gradually increased to the specified design rate, when the concrete was compacted. The complete centrifuging process of the specimens was performed in four stages. In Table 2 the experimentally based complete technological centrifugation process of the spun concrete members with the diameter of cross section of 560 mm is presented.

The curing of spun concrete was performed in the induction furnace and its total duration was 9 h: curing at ambient conditions (2 h); temperature increasing up to 75 °C (2 h); accelerated curing (3 h) and cooling to ambient temperature (2 h).

When conducting the research, the testing specimens were divided based on the effect of chemical admixtures on physical and mechanical properties of concrete: three-fourths of them had the same shape and included superplasticizers C-3 and ‘Dofen’, as well as resins ACF-3M, while the remaining specimens were the admixture-free control specimens.

**Figure 6:**
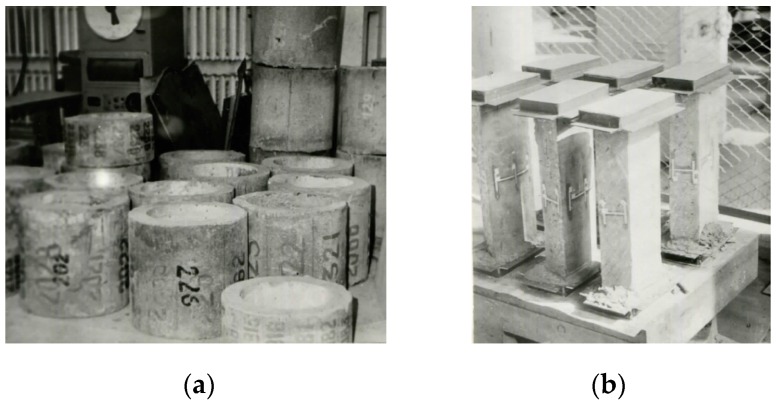
Spun concrete specimens for testing the effectiveness of chemical admixtures: (**a**) specimens of annular cross section and (**b**) cut out prism cores.

Sixty prism specimens of annular cross section cut out of spun members, twenty-four specimens of annular cross section and vibrated control cubes were used in testing the effect of chemical admixtures (Figure 6).

**Figure 7:**
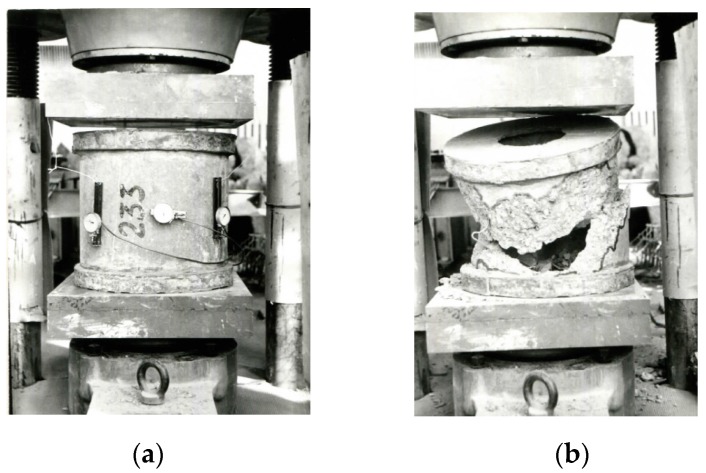
Spun concrete specimens of annular cross section: (**a**) before loading and (**b**) after loading.

**Figure 8:**
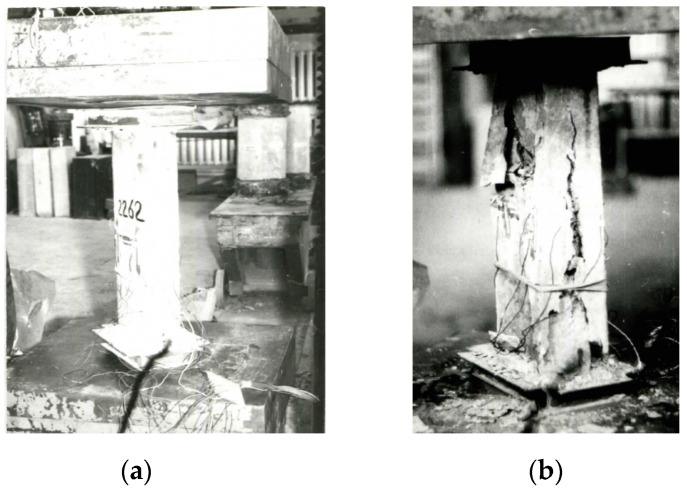
Cut out prism specimens of spun concrete: (**a**) before testing and (**b**) after testing.

Prism specimens and specimens of annular cross section were subjected to the action of the short-term axial compressive load. The specimens before and after the tests are shown in Figure 7 and Figure 8.

The statistical analysis of experimental data, evaluated by average, standard deviation (SD) and coefficient of variation (CV) is presented in Table 3. It should be noted, that increasing of coefficient of variation was caused by the fact that spun concrete specimens were made under factory conditions.

The test results confirm that the positive effect of admixtures on the mechanical properties of spun concrete was more pronounced in the case of vibrated concrete at its early age. Moreover, for spun concrete, this effect was less noticeable as compared to vibrated concrete and decreased with time. It was also determined that the effectiveness of the admixture ‘Dofen’, based on the strength of spun concrete, had decreased from 43% to 12% during the first 28 days [36].

**Figure 9:**
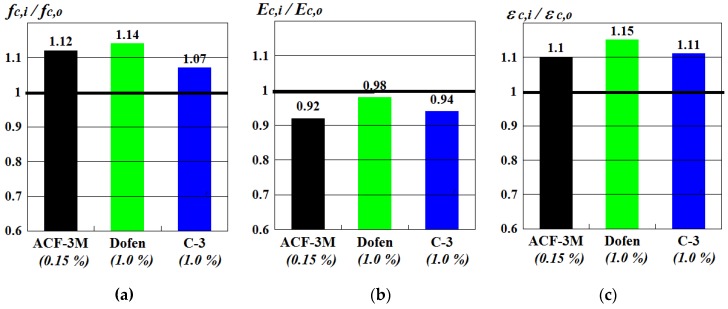
The ratios of: (**a**) prism strength; (**b**) initial modulus of elasticity and (**c**) longitudinal strains. Spun concrete, made using chemical admixtures ($f_{c,i}\;E_{c,i}\;e_{c,i}$) and without them ($f_{c,o}\;E_{c,o}\;e_{c,o}$), after 100 days of hardening.

The effect of chemical admixtures on the mechanical properties of ‘old’ (>100 days) spun concrete under axial compression was assessed using the dimensionless coefficient α (3), based on the results of a short-term compressive test performed on tubular spun specimens and prisms cut out of them. The main results of this testing, expressed in the parameters of prism strength ($f_{c,i}$) and stiffness ($E_{c,i}$, $\varepsilon_{c,i}$) are given in a histograms (Figure 9a–c).

Based on the data presented in Figure 9a, it could be concluded that spun concrete produced using superplasticizers ‘Dofen’ and C-3, as well as formaldehyde resin ACF-3M, had higher prism strength than regular spun concrete without admixtures. The level of positive effect of admixtures on the prism strength of spun concrete, which was more than 100 days old, was insignificant (within the usual bounds of the dispersion of the test results), depended on the admixture type and made about 10% on average. Thus, chemical admixtures had a much smaller positive effect on the strength of spun concrete than in the case of vibrated concrete. Moreover, the effect caused by admixtures during the production of spun concrete did not depend on their plasticizing properties. The insignificant effect of admixtures on the prism strength of spun concrete could be explained by the fact that compaction of the concrete mix in this case was rather specific as centrifugation allowed us to obtain concretes of the same density from mixes of various workability.

As can be seen from Figure 9b, superplasticizers ‘Dofen’ and C-3 and resin ACF-3M had an insignificant effect on the initial modulus of elasticity of spun concrete, which was more than 100 days old. It was confirmed by the fact that the ratios between the initial modulus of elasticity of concrete with an admixture $E_{c,i}$ and admixture-free concrete $E_{c,o}$ were close to unity and made $E_{c,i}/E_{c,o}=0.92;\;0.98;0.94$ on average.

Despite higher strength of spun concrete with an admixture (as compared to admixture-free concrete), it was significantly more deformable once it reached the age of 100 days. This was confirmed by the ratios of longitudinal strains of concrete with an admixture $\varepsilon_{c,i}$ and admixture-free concrete $\varepsilon_{c,o}$ given in Figure 9c.

Admixtures had a positive effect on the conditions of the concrete structure formation during its curing. This effect was evident not only from quicker formation of cement stone structure, but also from fewer microdefects, which normally emerge in concrete curing, in its newly formed structure. In loading ordinary concrete without any admixtures, more serious microdefects of its structure caused by curing and their later autogenous healing show trouble (fault) symptoms. Therefore, the strength of this concrete was lower than the strength of concrete made with admixtures. Moreover, admixtures increase the density of concrete and its resistance to aggressive environments.

## 4. Results and Conclusions

The results of the performed investigation allowed us to discuss them and draw the conclusions as follows:

1. The effectiveness of chemical admixtures depended on a number of technological factors. These factors might be considered the most important of them: a class of the concrete mix according to its workability and power intensity of its curing. The use of chemical admixtures for making spun concrete had some peculiarities, depending on the technologies of making (compacting) the elements, taking into consideration the fact that in centrifuging a part of chemical admixtures were washed out with the water. The effectiveness of chemical admixtures should be determined, depending on the particular type of concrete and the technology of its production.
2. The research results showed that using of such chemical admixtures as superplasticizers C-3, ‘Dofen’ and formaldehyde resin ACF-3M was an effective means of improving the properties of both vibrated and spun concrete. However, the superplasticizers ‘Dofen’ and C-3 had a more positive effect on the physical and mechanical properties of vibrated rather than spun concrete compared to the resin admixture ACF-3M. Using of C-3 admixture requires more strict technological conditions because this admixture reduces the time prior to the beginning of cement setting almost twice.
3. The positive effect of chemical admixtures for spun and vibrated concrete was most pronounced at an early age of concrete. Moreover, for spun concrete, this effect was less noticeable (by about 10%) as compared to vibrated concrete and did not depend on their plasticizing properties.
4. The effective amount of chemical admixtures for vibrated concrete was 0.1% of cement mass when formaldehyde resin ACF-3M and 0.6% of cement mass when superplasticizers C-3 and ‘Dofen’ were used. However, the appropriate amount of admixtures for spun concrete was larger, making, respectively, 0.15% of cement mass using formaldehyde resin ACF-3M and 1% of cement mass when superplasticizers C-3 and ‘Dofen’ were used. This was explained by the fact that about 25–30% of the amount of admixtures was washed out together with the water flowed out in the process of centrifugation.

## Figures and Tables

**Table 1:** Statistical analysis of experimental data of vibrated concrete specimens.

Admixture	Amount of Specim.	Prism Strength of Vibrated Concrete (f_c,i_)					
		After 3 Days of Hardening			After 5 Days of Hardening		
		Average (MPa)	SD (MPa)	CV (%)	Average (MPa)	SD (MPa)	CV (%)
Admixt. free	8	32.0	1.66	5.2	49.0	2.06	4.2
ACF-3M (0.1%)	8	38.0	2.20	5.8	52.0	2.70	5.2
Dofen (0.6%)	8	58.0	2.38	4.1	80,0	2.81	3.5
C-3 (0.6%)	8	51.0	1.94	3.8	72.0	3.03	4.2

**Table 2:** Centrifugation stages (when the diameter of the member d = 560 mm).

Centrifug. Stage	Spinning Rate (rev/min)	Pressure (MPa)	Spinning Time (min)	The Effect on the Concrete Mix
I	80–150	0.01	3–4	Concrete distrib. in the mold
II	200–250	0.03	1–2	Compaction
III	250–300	0.06	1–2	Compaction
IV	400–500	0.1–0.2	12–15	Compaction

**Table 3:** Statistical analysis of experimental data of spun concrete specimens.

Admixt.	Amount of Specim.	Prism Strength (f_c,i_)			Initial Modulus of Elasticity (E_c,i_)			Longitudinal Strains (ε_c,__i_)		
		Aver. (MPa)	SD (MPa)	CV (%)	Aver. (GPa)	SD (GPa)	CV (%)	Aver. 10^3^	SD 10^3^	CV (%)
Admixt. free	15	48.0	4.70	9.8	33.9	0.24	7.1	2.04	0.19	9.2
ACF-3M (0.15%)	15	53.7	4.89	9.1	31.1	0.19	6.2	2.24	0.21	9.5
Dofen (1%)	15	55.4	4.32	7.8	33.2	0.18	5.6	2.35	0.20	8.3
C-3 (1%)	15	50.9	3.31	6.5	32.2	0.22	6.8	2.27	0.18	8.0
